# Challenging Evaluation of Aortic Regurgitation: More Than a Quadricuspid Valve

**DOI:** 10.5935/abc.20180106

**Published:** 2018-07

**Authors:** Gonçalo Pestana, Carla Sousa, Teresa Pinho, Sara Maia, M. Júlia Maciel

**Affiliations:** Serviço de Cardiologia, Centro Hospitalar de São João, Porto - Portugal

**Keywords:** Aortic Valve Insufficiency, Echocardiography, Transesophageal, Echocardiography, Three-Dimensional, Pulmonary Disease, Chronic Obstructive

A 61-year-old female patient with chronic obstructive pulmonary disease, and no other comorbidities, was referred for cardiological assessment due to aggravated exertional dyspnea (New York Heart Association - NYHA class III) and atypical chest pain. Physical examination disclosed only a diastolic murmur in the second intercostal space in the right sternal border.

**Figure 1:**
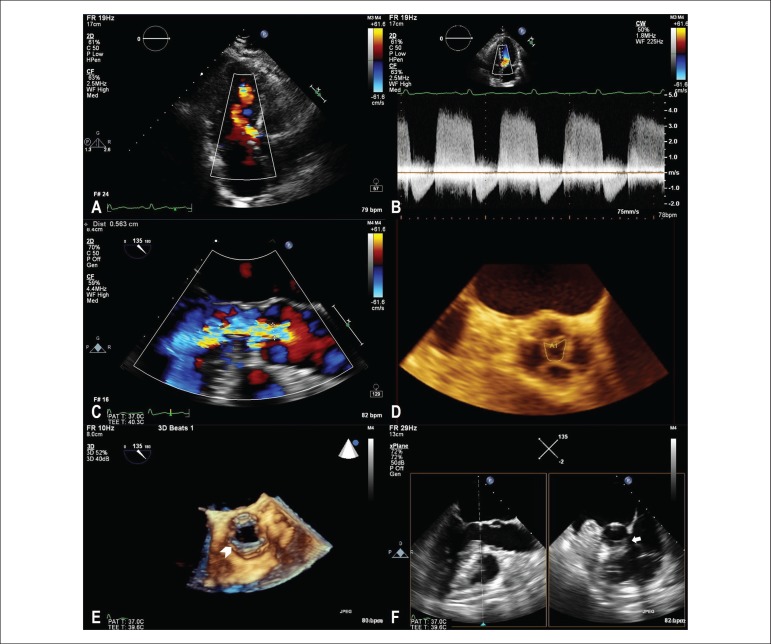
Aortic regurgitation jet visualized by transthoracic echocardiogram with color Doppler (A) and its respective spectrum on continuous Doppler wave (B); large jet visualized by transesophageal echocardiogram, with a 6 mm vena contracta (C), originating from the central coaptation defect of the quadricuspid aortic valve, with a regurgitant orifice of 0.35 cm^2^ in three-dimensional planimetry (D); Almost circumferential thickening of the left ventricular outflow tract readily identified in the three-dimensional image in systole (E), confirming the presence of a subaortic membrane with evaluation of orthogonal planes (F).

The transthoracic echocardiogram (TTE) showed aortic regurgitation (Figure 1A), with non-dilated cardiac chambers and preserved biventricular function. The acoustic window limited the evaluation of the valvular lesion severity and its importance in the context of the complaints, although the continuous Doppler spectrum suggested significant regurgitation (Figure 1B). The evaluation was further impaired by a systolic flow acceleration in the Left Ventricular Outflow Tract (LVOT), with no significant gradient and an undetermined cause. The aortic root had normal size, but it was not possible to carry out an adequate morphological and functional characterization of the valve.

The Transesophageal Echocardiogram (TEE) disclosed a quadricuspid aortic valve with central coaptation defect of 0.35 cm^2^ through three-dimensional planimetry causing severe aortic regurgitation (Figures 1C and 1D). The three-dimensional evaluation also showed a practically circumferential thickening in the LVOT, corresponding to a non-obstructive subaortic membrane, resulting in the observed flow acceleration (Figures 1E and 1F).

Medical therapy was optimized, and the patient was referred to valve replacement surgery.

This case highlights the incremental role of TEE, complemented by three-dimensional imaging, in the thorough assessment of valvular disease, crucial for the correct therapeutic management. This association between aortic quadricuspid valve and the subaortic membrane is a rare finding, described in only one previous report in the literature.^1^
